# Functional Imaging with ^18^F-FDG PET/CT and Diffusion Weighted Imaging (DWI) in Early Response Evaluation of Combination Therapy of Elotuzumab, Lenalidomide, and Dexamethasone in a Relapsed Multiple Myeloma Patient

**DOI:** 10.3390/diagnostics7040061

**Published:** 2017-12-15

**Authors:** Christos Sachpekidis, Antonia Dimitrakopoulou-Strauss, Stefan Delorme, Hartmut Goldschmidt

**Affiliations:** 1 Medical PET Group-Biological Imaging Clinical Cooperation Unit Nuclear Medicine, German Cancer Research Center, Im Neuenheimer Feld 280, D-69210 Heidelberg, Germany; a.dimitrakopoulou-strauss@dkfz.de; 2 Department of Internal Medicine V, University Hospital Heidelberg, D-69210 Heidelberg, Germany; Hartmut.Goldschmidt@med.uni-heidelberg.de; 3 Department of Radiology, German Cancer Research Center (DKFZ), D-69210 Heidelberg, Germany; s.delorme@dkfz-heidelberg.de; 4 National Center for Tumor Diseases (NCT) Heidelberg, D-69210 Heidelberg, Germany

**Keywords:** multiple myeloma, elotuzumab, lenalidomide, ^18^F-fluoro-2-deoxy-d-glucose positron emission tomography/computed tomography (^18^F-FDG PET/CT), diffusion weighted imaging (DWI)

## Abstract

Elotuzumab is the first monoclonal antibody approved for the treatment of relapsed-refractory multiple myeloma (MM) in combination with lenalidomide, an immunodulatory drug, and dexamethasone. We report on a multiply pre-treated MM patient with disease progression due to appearance of new focal lesions on imaging modalities, who was started on a combination treatment of elotuzumab, lenalidomide, and dexamethasone. After completion of three cycles of the new therapy the patient responded very well with a major decline of serological myeloma activity parameters serum monoclonal protein, kappa light chains, free light chains (FLC) ratio. The patient was also monitored with the functional imaging modalities ^18^F-FDG PET/CT and diffusion weighted imaging (DWI), which exhibited a mismatch of almost complete metabolic remission on positron emission tomography/computed tomography (PET/CT) with ^18^F-fluoro-2-deoxy-d-glucose (^18^F-FDG) (consistent with the serological response), and signal elevation persistence on DWI. This case demonstrates the potentially superior performance of ^18^F-FDG PET/CT over DWI in early response evaluation of combined treatment with a monoclonal antibody, an immunomodulatory drug, and dexamethasone in MM.

**Figure 1:**
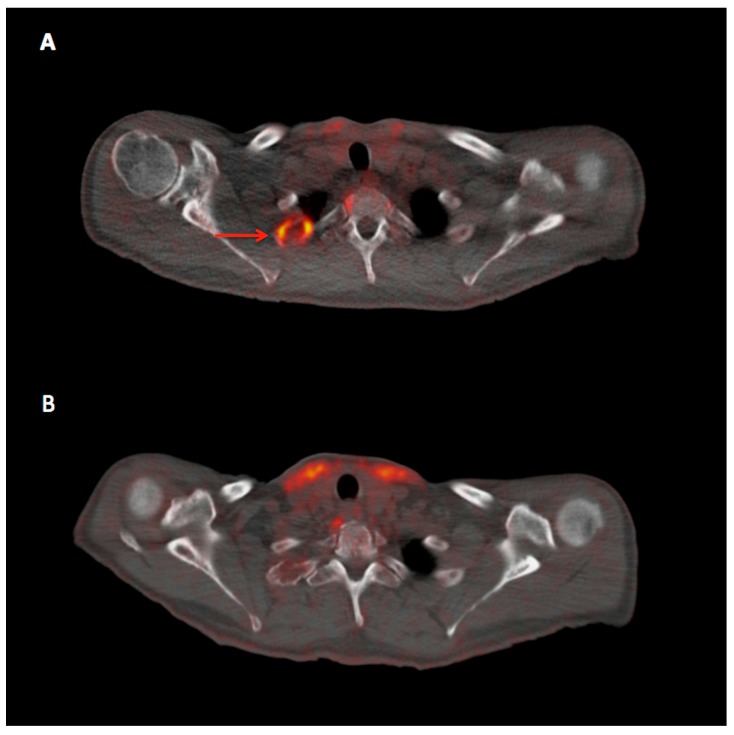
Transaxial fused ^18^F-fluoro-2-deoxy-d-glucose positron emission tomography/computed tomography (^18^F-FDG PET/CT) images of the relapsed 73-year-old multiple myeloma (MM) patient before (**A**) and after (**B**) three cycles of combination treatment with elotuzumab, lenalidomide, and dexamethasone. The patient demonstrated, among others, a highly ^18^F-FDG avid, osteolytic lesion in the second rib on the right before initiation of the new treatment (arrow, **A**), which responded with an almost complete metabolic remission after three cycles (B). The previously thrice treated with high-dose chemotherapy and autologous stem cell transplantation patient showed a major decrease of the serological myeloma activity parameters as response to the combined treatment: serum monoclonal protein decreased from 4.6 g/L to detectable but not quantifiable levels, kappa light chains decreased from 37.6 mg/L to 22.7 mg/L, and the free light chains (FLC) ratio was normalized from 3.2 to 1.6. Thus, the imaging findings on follow-up PET/CT were considered in line with the serological response. Elotuzumab is a humanized immunostimulatory antibody that recognizes lymphocytic activation molecule F7 (SLAMF7), a surface glycoprotein expressed by myeloma and natural killer cells, and elicits a dual effect by directly activating natural killer cells and mediating antibody-dependent cell-mediated cytotoxicity via the CD16 pathway to cause targeted myeloma cell death [1]. Elotuzumab is the first monoclonal antibody approved for the treatment of relapsed-refractory MM in combination with lenalidomide, an immunodulatory drug, and dexamethasone. The combination of elotuzumab, lenalidomide, and dexamethasone leads to an improved progression-free survival, overall response rate and interim overall survival in patients with relapsed or refractory MM, as compared to the standard regiment of lenalidomide and dexamethasone [2,3].

**Figure 2:**
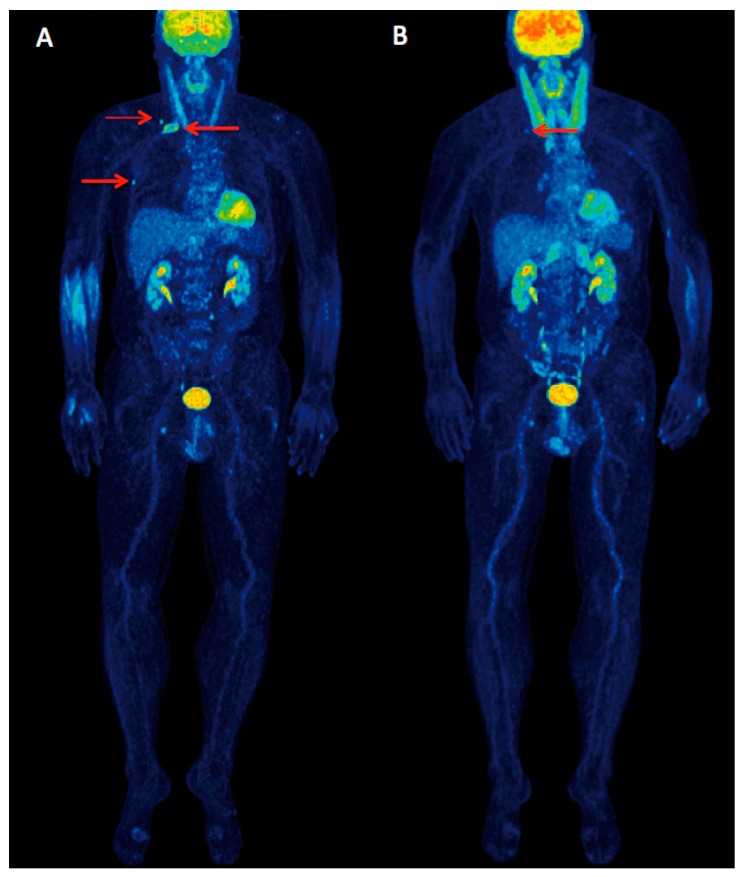
Maximum intensity projection (MIP) ^18^F-FDG PET/CT images before (**A**) and after three cycles of combination treatment with elotuzumab, lenalidomide and dexamethasone (**B**). The PET/CT scan before treatment showed three foci of increased ^18^F-FDG uptake in the right scapula, the second rib and the sixth rib (arrows, **A**) as well as moderate diffuse bone marrow tracer uptake in the spine and pelvis. Follow-up PET/CT scan after three cycles of the combined treatment showed complete remission of the metabolic activity in the right scapula and the sixth rib, and only faint tracer uptake in the second rib, which represented the previously most metabolically active myeloma lesion (arrow, **B**). Moreover, a decline of the bone marrow tracer uptake was observed and confirmed by a decrease of the respective SUV values (for example a decrease in the left iliac bone maximum standardized uptake value (SUV_max_) from 2.5 before therapy to 1.6 after three cycles of therapy). The patient also underwent dynamic PET/CT scanning of the pelvis, which revealed a decrease of the ^18^F-FDG-associated kinetic parameters K_1_, influx, and fractal dimension in the bone marrow of the iliac bone. It has been proven that SUV values and kinetic parameters of ^18^F-FDG correlate positively with bone marrow plasma cell infiltration rate [4]. Moreover, a full kinetic analysis of the ^18^F-FDG PET studies prior and after the first cycle of anthracycline-based chemotherapy in patients with MM has been proven helpful for the prediction of progression-free survival [5]. The accurate and early assessment of the depth of treatment response is of paramount importance in the MM workup. ^18^F-FDG PET/CT represents the best imaging tool for treatment monitoring in MM with a prognostic role both at diagnosis and after treatment [6,7,8]. It has been recently proposed that a combination of a negative bone marrow assay, a negative PET/CT, and a normal serum FLC ratio may reflect the complete eradication of MM cells from all compartments [6].

**Figure 3:**
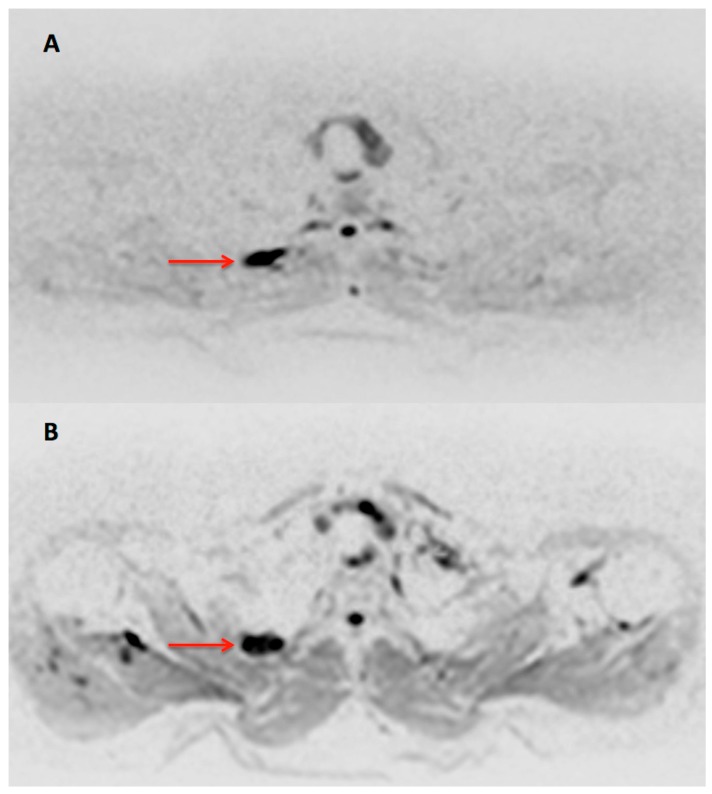
Transaxial DWI b800 (black/white inverted) images before (**A**) and after (**B**) three cycles of the combination treatment. DWI demonstrated a persistent signal elevation in the osteolytic lesion of the second rib (arrow) after the administration of treatment, despite the patient’s serological response. Although existing data are rather limited, DWI is considered a repeatable, quantifiable magnetic resonance imaging-technique that can potentially help differentiate responders from nonresponders in myeloma bone disease [9,10,11]. In an study involving a mixed population of primary and pre-treated MM patients DWI was found to be more sensitive than ^18^F-FDG PET in detecting myeloma lesions, when using the well-established imaging standard of MRI and/or CT as reference. However, this mismatch between PET and DWI findings was mainly seen in the already treated patients, who had reached at least near complete response, raising, thus, the suspicion of false-positive findings on DWI [12]. A possible explanation is the fact that diffusion imaging is based on T2-weighted sequences, and diffusion weighting is introduced using de- and rephrasing preparation gradients. As a result, strongly T2-hyperintense tissue will appear hyperintense on plain DWI-images (T2-shinethrough), as it was in the presented case. After successful treatment, solid myeloma foci often develop high signal intensity on T2-weighted images, which explains why DWI may be false positive in pre-treated myeloma. The problem can theoretically be overcome by calculating the apparent diffusion coefficient (ADC) from at least two DWI sequences using different preparation gradients. ADC maps, however, are often degraded by artefacts in areas with magnetic field inhomogeneities and respiratory motion. This was the case here, where the lesion was located in the upper thoracic aperture. On the other hand, in that study the two functional modalities (PET, DWI) were equally sensitive in the sub-population of primary, untreated MM patients [12]. This is the first case demonstrating the potentially superior performance of ^18^F-FDG PET/CT over DWI in early response evaluation of combined treatment of a monoclonal antibody, an immunomodulatory drug and dexamethasone in MM. Further prospective studies focusing on the direct comparison between PET and DWI in MM treatment response evaluation are required to elucidate this topic.
